# Exotic *Leucaena leucocephala* cultivates distinct rhizosphere soil microbial communities from native *Albizia kalkora* in a dry-hot valley of China

**DOI:** 10.3389/fmicb.2026.1866484

**Published:** 2026-07-08

**Authors:** Xuemei Wang, Yingyan Wang, Tianzhi Huang, Bangguo Yan, Jixia Zhao, Gangcai Liu

**Affiliations:** 1 School of Geography and Environment, Mianyang Normal University, Mianyang, Sichuan, China; 2 School of Resources and Environment, Baoshan University, Baoshan, Yunnan, China; 3 College of Resources and Environment, Yunnan Agricultural University, Kunming, China; 4 Key Laboratory of Mountain Surface Processes and Ecological Regulation, Institute of Mountain Hazards and Environment, Chinese Academy of Sciences and Ministry of Water Resources, Chengdu, China

**Keywords:** bacteria, fungi, invasive plant, legumes, microbial diversity

## Abstract

Rhizosphere microbes play a critical role in plant growth and invasiveness, and plants significantly impact their rhizosphere microbial communities. Legumes are closely related to soil microorganisms; however, the impact of native and exotic legumes on rhizosphere soil microbial communities is not fully understood. The objective of this study was to compare the differences of the rhizosphere microbial communities between native *Albizia kalkora* and non-native *Leucaena leucocephala* in a co-established site in Yuanmou dry hot valley of China. Rhizosphere bacterial and fungal community composition, diversity, and soil enzyme activities of *A. kalkora* and *L. leucocephala* were analyzed via *in situ* sampling and a laboratory cultivation experiment. Results showed that there were significant differences in the composition of bacterial communities, and the bacterial alpha diversity of *L. leucocephala* was significantly lower than that of *A. kalkora* both *in situ* sampling and cultivation conditions. Although the composition of soil fungal community showed significant differences in situ sampling, no significant changes occurred in soil fungal community composition and diversity after 80 days of cultivation; but the β-glucosidase activity, arbuscular mycorrhizal fungi (AMF) abundance and amino acid metabolism abundance in *L. leucocephala* soil were higher than those in *A. kalkora* soil. Soil properties were the important factors influencing soil bacterial communities, but no correlation was found between fungal communities and soil properties under the same condition. These findings suggest that the invasive *L. leucocephala* rapidly establishes a distinct rhizosphere microbial community during early establishment that may confer a competitive advantage, which highlights the necessity of exploring the associated feedback effects to unravel the mechanisms of plant invasion.

## 1 Introduction

Soil microorganisms are the core driving force of soil fertility, which play an essential role in soil evolution, litter decomposition, and nutrient cycling (Bardgett and van der Putten, 2014). Bacteria and fungi dominate terrestrial soil habitats in terms of biodiversity, biomass and their influence over essential soil processes (Bahram et al., 2018). Plants exert directed selection pressure on rhizosphere microbial communities through root exudates, litter input and symbiotic relationships (Bhatnagar et al., 2018; Hu et al., 2018), and they also indirectly affect microbial communities by affecting soil resource availability (Bais et al., 2006).

Difference in plant origin can lead to significant differentiation of rhizosphere microbial communities, which is particularly complex between native and exotic plants, especially the exotic invasive plants (Shelby et al., 2016). A growing number of studies focused on the effects of invasive plants on soil microbial communities (Gaggini et al., 2018; Zhu et al., 2020), as this represents one of the key mechanisms underlying successful invasion (Duchesneau et al., 2021). Exotic plant species commonly escape regulation by natural microbial enemies (Busby et al., 2016; McGinn et al., 2018) and frequently benefit substantially from mutualisms with soil biota (Zhang et al., 2010; Busby et al., 2016). Consequently, most studies have reported significant differences in the composition and diversity of soil microbial communities between native and exotic species (Gaggini et al., 2018; Ning et al., 2021). However, some studies have found no significant difference in soil bacterial or fungal communities between native and non-native species (Bickford et al., 2020; Zhu et al., 2020; Tian et al., 2025). Similarity, while plant invasion often reduces soil microbial alpha diversity (Gaggini et al., 2018; Jurkšienė et al., 2020; Zhu et al., 2020), higher microbial diversity in association with invasive species has also been observed (Song et al., 2017). Therefore, invasive plants affect the soil biota differentially (Torres et al., 2021), and no consistent conclusion has yet been reached.

Among many plant families, legumes play a key role in nutrient cycling and soil health in terrestrial ecosystems due to their symbiotic N fixation with rhizobium (Thilakarathna and Raizada, 2017). Legumes are not only symbiotic with N-fixing rhizobia, but symbiotic with P-acquiring arbuscular mycorrhizal fungi (AMF; van der Heijden et al., 2016), which are closely related to soil microorganisms. Legumes release flavonoids that attract rhizobia, uncharacterized compounds that induce branching of mycorrhizal hyphae, and arabinogalactans that trigger biofilm formation of specific beneficial bacteria (Sasse et al., 2018). Legumes can also affect soil nutrients through symbiotic nitrogen fixation, thus driving the succession and functional differentiation of microbial communities (Sprent, 2009). The interaction between legumes and rhizobia is locally evolved, and invasion can disrupt native belowground mutualisms and reduce native legumes fitness (Rodríguez-Echeverría et al., 2012). Current studies have characterized the microbial communities of invasive legume in their native and non-native ranges (Birnbaum et al., 2014, 2016; Shelby et al., 2016), and additional researches have also compared microbial communities between native and invasive legume species within the same region (Boukhatem et al., 2012; Busby et al., 2016; La Pierre et al., 2017; Muema et al., 2022). However, these comparative studies have focused predominantly on bacterial communities, particularly nodule-associated rhizobia, while the responses of fungal communities have received far less attention.

The native *Albizia kalkora* and exotic *Leucaena leucocephala* (both *Leguminosae*, *Mimosoideae*) are two dominant legume species in the dry hot valleys of southwest China, serving as phenetic counterparts that share similar morphological traits. Indigenous to Mexico and Central America, *L. leucocephala* is known to benefit dry forest restoration due to its fast growth rate, high drought tolerance, and good recovery following burning (Wolfe and Van Bloem, 2012; Sharma et al., 2022). *L. leucocephala* was introduced to Hainan Island, China, in 1961 and is now widely cultivated across southern China, serving as a key pioneer afforestation species in Yuanmou dry-hot valley of Yunnan (Zong et al., 2007). Its strong seed reproductive capacity and photosynthetic adaptability enable *L. leucocephala* to thrive under the harsh conditions of the dry-hot valley (Zong et al., 2007; Li et al., 2020). However, it is also recognized as a global invasive species that threatens the biodiversity in tropical and subtropical forests worldwide (Richardson and Rejmánek, 2011). Previous studies have provided insights into the belowground traits of these two species. Li and Zhao (2005) detected abundant AMF spores in the rhizosphere soil of both *A. kalkora* and *L. leucocephala*, with higher spore density recorded for *L. leucocephala*. Tang et al. (2012) reported that the nitrogenase activity of root nodules was higher in *L. leucocephala* than that in *A. kalkora*. These findings suggest that the microbial communities associated with *A. kalkora* and *L. leucocephala* may differ. However, to date, no studies have directly characterized the rhizosphere microbial communities of these two species.

Therefore, to investigate whether *A. kalkora* and *L. leucocephala* assemble distinct rhizosphere microbial communities, we adopted a two-step approach. We first conducted an *in situ* sampling at a site where *A. kalkora* and *L. leucocephala* co-occur to capture the field-based differences in rhizosphere microbial communities between the two species. Recognizing that field conditions may be confounded by underlying environmental heterogeneity (e.g., the surrounding bulk soil microbial pool), we then performed a controlled laboratory cultivation experiment using homogenized soil to determine whether the two plant species truly harbor distinct rhizosphere microbial communities. We hypothesized as follows:

*H1*: The rhizosphere soil microbial communities will differ significantly between the two legumes, with the exotic *L. leucocephala* exhibiting lower microbial diversity.

*H2*: Under cultivation conditions, the two plant species will selectively recruit the dominant and enriched microorganisms observed under field conditions, thereby re-establishing the distinct microbial community profiles detected in situ.

**Figure 1:**
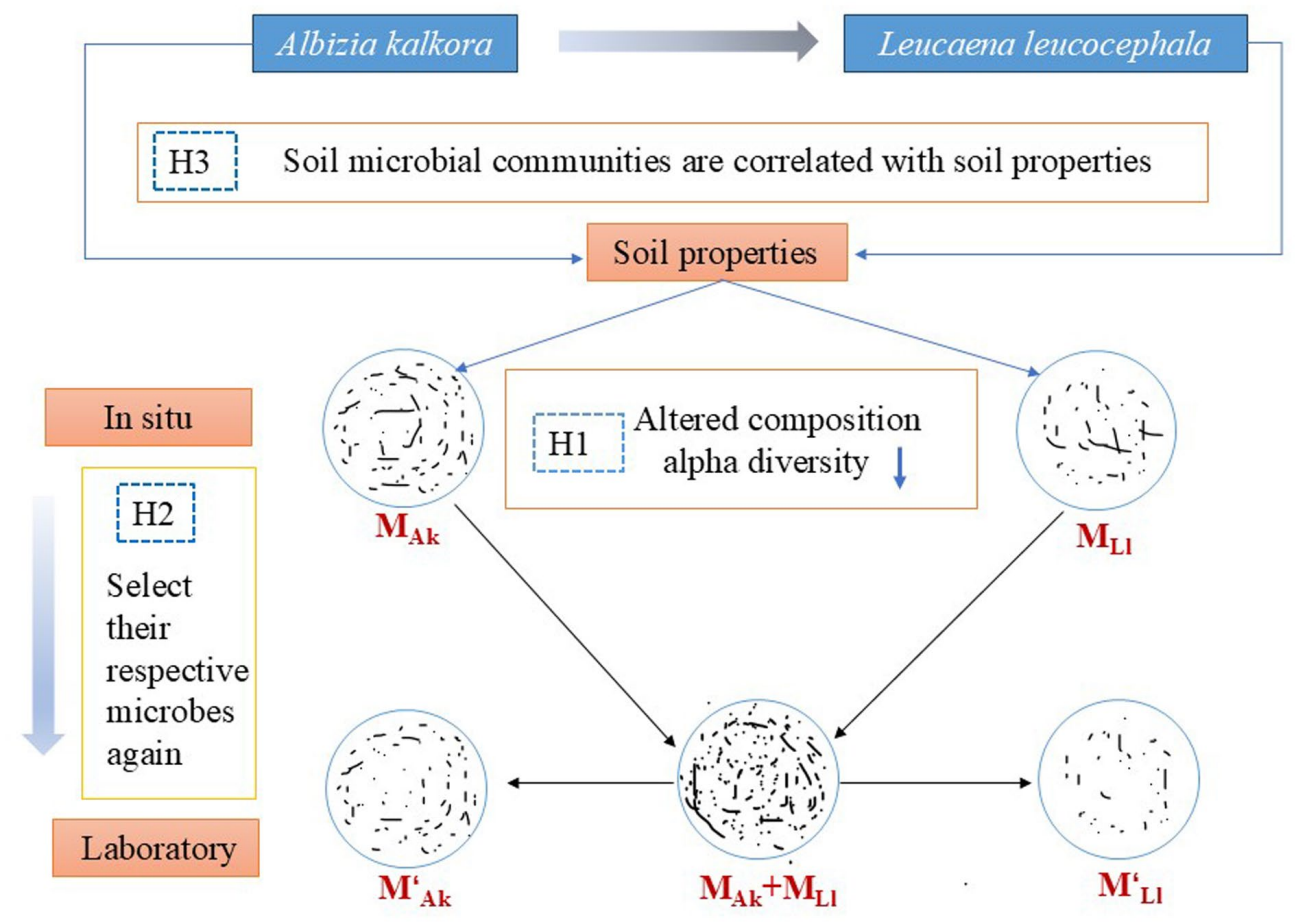
Hypothesis framework linking the effect of *Albizia kalkora* and *Leucaena leucocephala* on rhizosphere soil microbial communities. M_Ak_, microbial communities of *A. kalkora*; M_Ll_, microbial communities of *L. leucocephala*.

*H3*: Rhizosphere microbial communities will show significant correlations with soil properties under in situ conditions, while these correlations will be weakened or absent under laboratory cultivation conditions (Figure 1).

Testing these hypotheses may generate information to help understand the invasion mechanism of exotic legumes by regulating their relationship with soil microorganisms.

## 2 Materials and methods

### 2.1 Study site

The study site was located in the Yuanmou dry hot valley in Yuanmou County (101°35′–102°05′E, 25°25′–26°07′N), southwest China. Yuanmou dry hot valley is in the lower reaches of the Jinsha River, which is the upper reach of the Yangtze River. The area is characterized by an arid climate and barren soil, resulting in an extremely fragile ecological environment. The climate features pronounced dry and wet seasons. The wet season lasts from June to October, during which time the precipitation is 623.95 mm, accounting for more than 90% of the total rainfall. The dry season is very long and lasts up to 6–7 months (November to May). The soil type here is dry red soil, which is classified as Luvisols in FAO classification. The vegetation is classified as valley-type savanna, dominated by *Heteropogon contortus*, *Eulaliopsis binata*, *Bothriochloa pertusa*, *Cymbopogon goeringii* and *Dichanthium annulatum* (Yan et al., 2016, 2020). *A. kalkora* and *L. leucocephala* are commonly used as the main afforestation species for vegetation restoration in this area. *A. kalkora* is native to the dry hot valley, whereas *L. leucocephala* is an exotic species introduced for afforestation in Yuanmou in the 1990s. In the following decades, *L. leucocephala* spread naturally, growing rapidly and colonizing farmlands and roadsides in dry hot valleys, thereby posing a potential invasion risk.

### 2.2 *In situ* sampling

**Figure 2:**
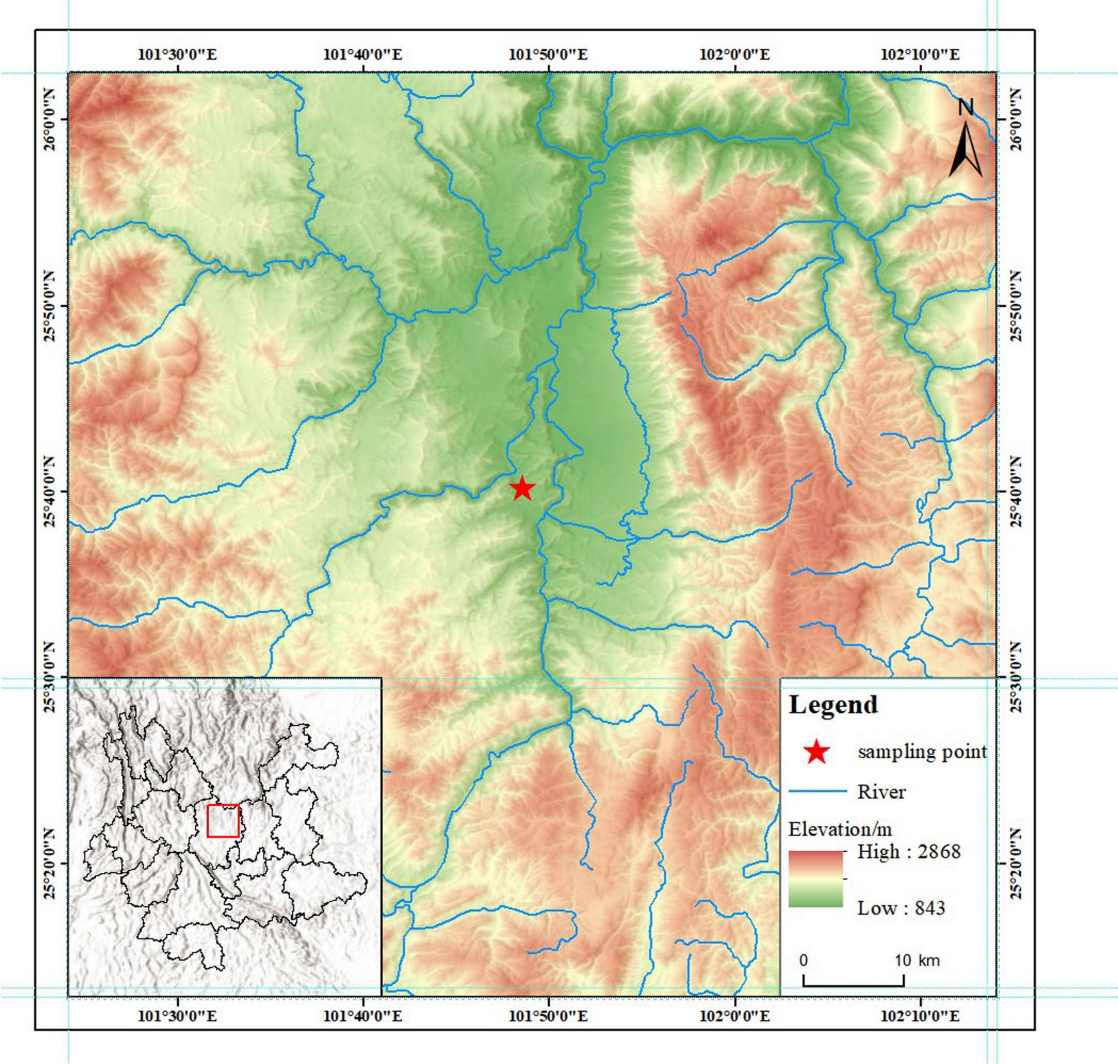
The sampling location in this study area.

Most individuals of *A. kalkora* and *L. leucocephala* were patchily distributed, meaning that variations in topography and soil properties across different locations could potentially influence their rhizosphere microbial communities. To minimize this confounding effect, we selected a single co-established site for both species in Dayibao (101.81°E, 25.67°N), Yuanmou County, Yunnan Province (Figure 2). This site originally served as a natural habitat for *A. kalkora*. Following the natural spread of *L. leucocephala* into this location, the two species have become co-established. Consequently, the environmental conditions were consistent and both *A. kalkora* and *L. leucocephala* naturally grew here. Six *A. kalkora* and *L. leucocephala* with basically the same size were selected for sampling. The growth information of *A. kalkora* and *L. leucocephala* was shown in Supplementary Table S1, and most of them were small shrubs. When sampling, after removing the surface soil and gently digging out the plants out of the soil, the roots were shaken to remove loosely associated soil, and the soil left on the roots was collected with a brush and tweezer. The tools were sterilized with 70% ethanol in-between sample collections. Then the rhizosphere soil samples were put in a sterilised bag, immediately placed on ice and transported to the laboratory, where they were stored at −20 °C until DNA extraction. Besides, due to the small amount of rhizosphere soil collected, loosely associated soil of each plant was also sampled for physicochemical parameters measurement and laboratory cultivation experiment.

Soil pH, soil organic matter, total N, total P, total K, available N, available P, and available K were measured according to the standard methods recommended by the Chinese Society of Soil Science (Lu, 2000). Besides, *β*-glucosidase was determined by nitrophenol colorimetry, and activity was expressed by the content of p-nitrophenol produced per unit time and unit of dry soil. Urease was determined by a colorimetric method using sodium phenol-sodium hypochlorite, and activity was expressed as mass of NH_4_^+^-N produced per unit time and unit dry soil. Acid phosphatase was estimated by measuring the release of p-nitrophenol from p-nitrophenyl phosphate (Sinsabaugh et al., 1999).

### 2.3 Laboratory cultivation experiment

The collected bulk soil was air-dried and sieved (2 mm), respectively, and equal amounts of *A. kalkora* and *L. leucocephala* soils were thoroughly mixed and used as potting soil, which contained both the soil microorganisms of *A. kalkora* and *L. leucocephala*. To explore the role of *A. kalkora* and *L. leucocephala* in selecting and shaping rhizosphere microbial communities when starting from a mixed microbial pool, *A. kalkora* and *L. leucocephala* were cultivated in potting soil, thus examining the short-term differences of the rhizosphere microbial community between the two legumes. Because the pot experiment began with a 1:1 mixture of soils from both species, any differences observed between the two species at the end of the experiment can be attributed to species-specific effect rather than initial bulk soil microbial pool. Plump seeds of *A. kalkora* and *L. leucocephala* were soaked in 98% sulfuric acid for 5 min to break the physical dormancy, and then they were washed with tap water repeatedly. After breaking the seed dormancy, 10 seeds of *A. kalkora* and *L. leucocephala* were sown in the corresponding pots, and then the pots were watered to keep the soil moist. All pots were placed in a climate chamber for cultivation, and the temperature of the climate chamber was set to 35 °C/25 °C (day/night: 16 h/8 h), which was close to the ambient temperature of the growing season in Yuanmou dry hot valley. After most seeds germinated, seedlings were thinned, and four were left in each pot. Each pot was watered with the same amount of water regularly, and the position of the pots in the climate chamber was moved regularly (up and down, side-to-side randomly).

The 80-day cultivation period was designed to capture the early phase of rhizosphere microbial community assembly following plant establishment, a critical window for understanding initial plant-driven selection. According to the growth and development of the plants, after 30 days of cultivation, *L. leucocephala* began to produce dead leaves, and after 50 days of cultivation, the height and nodule numbers of *A. kalkora* and *L. leucocephala* showed significant differences. After 80 days of cultivation, the dead leaves and the nodule numbers of *L. leucocephala* increased significantly (Supplementary Table S2). Therefore, refer to other relevant studies (Marschner et al., 2002), three pots of *A. kalkora* and *L. leucocephala* were destructively harvested after 30 days and 50 days, and the remaining plants continued to grow. After 80 days of cultivation, the experiment was finished, and six pots of *A. kalkora* and *L. leucocephala* were destructively harvested. When harvested, roots from each pot were excavated carefully, and the rhizosphere soil attached to the root system was shaken off and collected with a brush. Apart of soil was stored at −20 °C for determining soil microbial community, and another part was stored at 4 °C for determining soil organic matter, soil available N, available P and soil enzyme activities.

### 2.4 DNA extraction, amplification, sequencing and bioinformatics analysis

About 0.5 g of fresh soil was weighed from each sample, and the total DNA was extracted using the Mobio Powersoil DNA Isolation kit (Mobio Laboratories, Carlsbad, CA, United States) according to the manufacturer’s instructions. Extracted soil DNA was quantified using a NanoDrop ND-2000c UV–Vis spectrophotometer (NanoDrop Technologies, Wilmington, DE, United States), and the quality of DNA was detected using 1.2% agarose gel electrophoresis. A paired-end dual-index sequencing approach was used. Bacterial 16S rRNA genes (v3-v4) were amplified by PCR using the primers 338F (5′-ACTCCTACGGGAGGCAGCA-3′) and 806R (5′-GGACTACHVGGGTWTCTAAT-3′; Claesson et al., 2009). The fungal ITS gene (ITS1) was amplified using ITS1F (5′-CTTGGTCATTTAGAGGAAGTAA-3′) and ITS2 (5′-GCTGCGTTCTTCATCGATGC-3′; Orgiazzi et al., 2012). The amplification procedure was as follows: 95 °C for 2 min; 25 cycles at 95 °C for 30 s, 55 °C for 30 s, 72 °C for 30 s; and a final extension at 72 °C for 5 min. Three replicate PCRs were carried out for each DNA sample, and subsequent products were pooled together. The PCR products were confirmed using agarose gel electrophoresis, subsequently isolated from the gel, and purified using a Gene JET gel extraction kit (Thermo Fisher Scientific, Waltham, MA, United States). The standard Illumina Truseq DNA library preparation process was used to construct the required genomic library. After the library was qualified, purified PCR products were sequenced using an Illumina MiSeq platform (Illumina Inc., San Diego, CA, United States) at Shanghai Personal Biotechnology Co., Ltd. (Shanghai, China).

Microbial sequences were processed using the “Quantitative insights into microbial ecology 2 (QIIME2) (2019.4).” Reads containing ambiguous bases were discarded; only overlapping sequences longer than 10 base pairs were assembled. After sequence optimization and data quality control, microbial sequence data were processed using the amplicon sequence variants (ASV) method in QIIME2 and DADA2. The taxonomy of each 16S rRNA and ITS gene sequence was analyzed using the Silva Database (version 132; Quast et al., 2013) and the Unite Database (version 8.0; Kõljalg et al., 2013), respectively. A total of 4,039,418 and 3,692,728 raw sequences (average 112,206 and 102,576 per sample) were obtained for bacteria and fungi, respectively. After quality filtering, trimming, and chimera removal, 3,421,589 and 3,310,797 high-quality sequences remained, respectively. To account for the bias caused by uneven sequencing depth, each sample was rarefied to the smallest library size, resulting in 58,409 sequences per sample for the 16S rRNA gene and 51,205 sequences per sample for the ITS1 region. Taxonomic alpha diversity of soil bacteria and fungi was estimated using Chao1, Pielou’s evenness, Shannon-Wiener, and Simpson diversity indices.

### 2.5 Statistical analysis

Independent-samples *t*-test was used to test the significant difference for soil parameters *in situ* sampling. The Mann–Whitney U test followed by Benjamini-Hochberg correction was used to assess the differences for alpha diversity indices and predicted functions between the rhizosphere soil of *A. kalkora* and *L. leucocephala*. Permutational multivariate analysis of variance (PERMANOVA using the adonis2 function in the vegan package in R 4.4.0) was used to analyse the effects of plant, time, and their interaction on rhizosphere soil bacterial and fungal community structures under cultivation condition. Besides, spearman correlation analysis and redundancy analyses (RDA) were employed to analyze the correlation between soil microbial communities and soil chemical parameters, and mantel tests were used to test the correlations between soil parameters and bacterial and fungal communities under the same condition.

Analysis of similarities (ANOSIM) based on the Bray–Curtis dissimilarity matrix was performed to identify whether there were significant differences between the two legumes, and non-metric multidimensional scaling (NMDS) analysis was performed to visualize the differences of the microbial communities among different treatments using the ‘Vegan’ package in R. Significant differences in taxa (six taxonomic levels from phylum to species) of soil microbial communities between the two legumes were further analyzed using linear discriminant analysis (LDA) effect size (LEfSe) analysis.^1^

Furthermore, bacterial community functions were predicted by PICRUSt2 (Langille et al., 2013). The Kyoto Encyclopedia of Genes and Genomes (KEGG) database was used to identify the functional genes, and functional predictions were produced from the KEGG database using 16S rRNA data. The tool FUNGuild^2^ was used to analyze the functional classification of fungi based on high-throughput sequencing data. FUNGuild classified the trophic modes of fungi into three types: (1) pathotroph = receiving nutrients by harming host cells (including phagotrophs); (2) symbiotroph = receiving nutrients by exchanging resources with host cells; and (3) saprotroph = receiving nutrients by breaking down dead host cells. Within these trophic modes, a total of 12 categories were designated as guilds (in alphabetical order: animal pathogens, AMF, ectomycorrhizal fungi, ericoid mycorrhizal fungi, foliar endophytes, lichenicolous fungi, lichenized fungi, mycoparasites, plant pathogens, undefined root endophytes, undefined saprotrophs, and wood saprotrophs; Nguyen et al., 2016). The confidence level “possible” was deleted, and only “highly probable” and “probable” were included.

## 3 Results

### 3.1 The alpha diversity of rhizosphere soil bacterial and fungal communities of the two legumes

**Figure 3:**
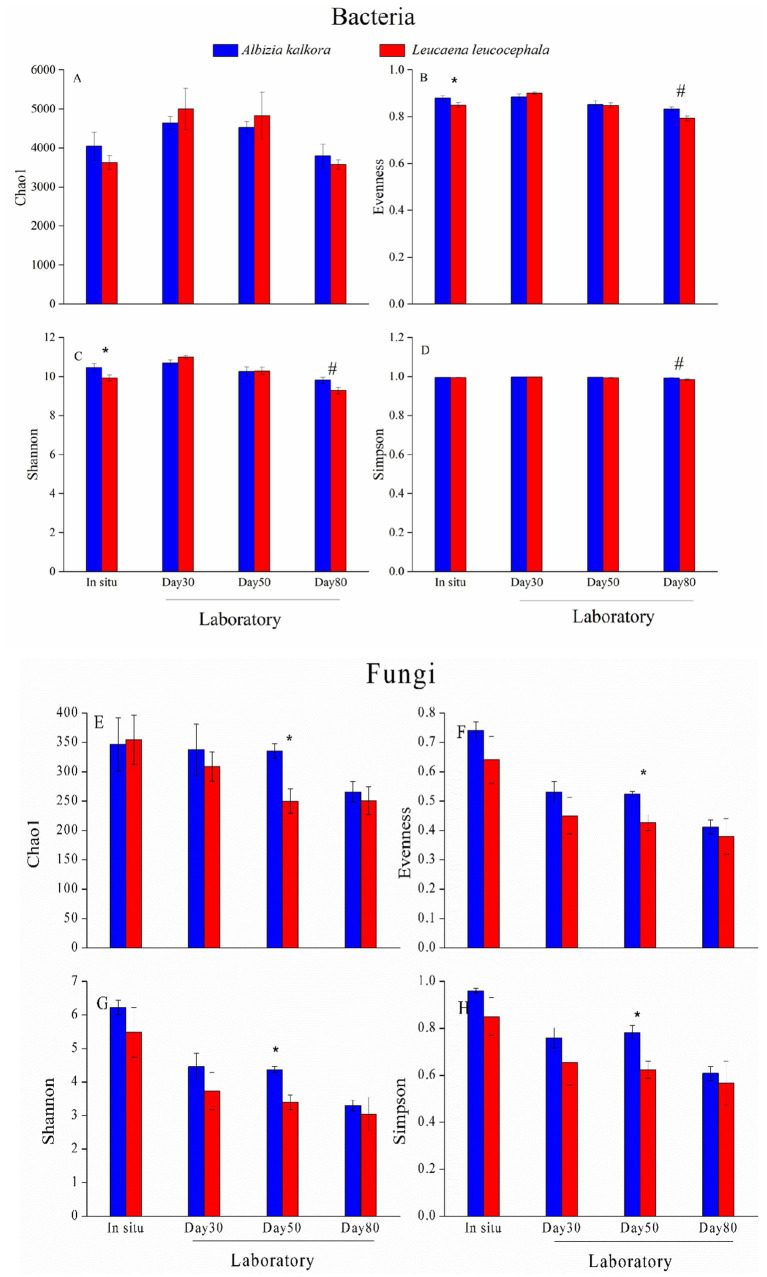
Effect of plant treatments on alpha diversity indices of rhizosphere soil bacterial **(A–D)** and fungal **(E–H)** communities. *p*-values were derived from the Mann–Whitney U test with Benjamini-Hochberg false discovery rate adjustment for multiple comparisons. #*p* < 0.1, *p < 0.05, **p < 0.01, ***p < 0.001.

*In situ* sampling, the bacterial Evenness and Shannon indices of *L. leucocephala* were 3.4 and 5.2% lower than those of *A. kalkora*, respectively (*p* = 0.05), but the fungal alpha diversity indices of *L. leucocephala* and *A. kalkora* had no significant difference. Under cultivation conditions, with the increased cultivation time, Chao1, Evenness, Shannon and Simpson indices of bacterial and fungal community decreased. For soil bacterial community, after 80 days of cultivation, the Evenness, Shannon and Simpson indices of *L. leucocephala* were 4.8, 5.4 and 1.0% lower than those of *A. kalkora*, respectively, with the corrected *p* value indicated a marginally significant difference (*p* < 0.1). Significant differences in fungal alpha diversity between the two legumes were detected only after 50 days (*p* = 0.05), while no such differences were observed after 30 or 80 days (Figure 3; Supplementary Table S3).

### 3.2 The beta diversity of rhizosphere soil bacterial and fungal communities of the two legumes

**Figure 4:**
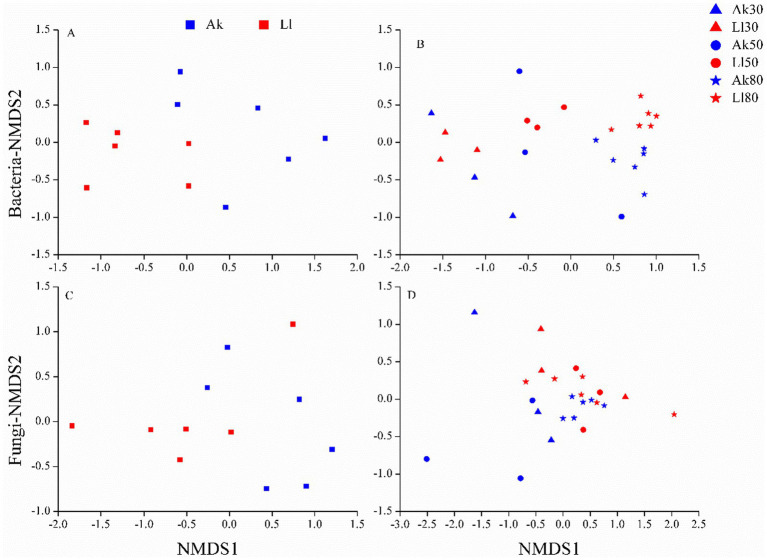
Plots of nonmetric multidimensional scaling (NMDS) based on Bray-Curtis distance of rhizosphere soil bacterial and fungal communities. **(A)** rhizosphere soil bacterial communities under *in situ* sampling; **(B)** rhizosphere soil bacterial communities in laboratory cultivation experiment; **(C)** rhizosphere soil fungal communities under in situ sampling; **(D)** rhizosphere soil fungal communities in laboratory cultivation experiment. Ak, *Albizia kalkora*; Ll, Leucaena leucocephala; Ak30: cultivate by *Albizia kalkora* after 30 days; Ak50: cultivate by *A. kalkora* after 50 days; Ak80: cultivate by *A. kalkora* after 80 days; Ll30: cultivate by *Leucaena leucocephala* after 30 days; Ll 50: cultivate by *L. leucocephala* after 50 days; Ll 80: cultivate by *L. leucocephala* after 80 days.

Both bacterial and fungal community structures were clustered in the NMDS ordination. ANOSIM analysis revealed that the two legumes affected bacterial (*r* = 0.443, *p* = 0.013) and fungal (*r* = 0.343, *p* = 0.015) community structure *in situ* sampling (Figure 4). Under cultivation conditions, PERMANOVA showed that plant, time and their interaction had significant effects on soil bacterial communities; plant had no significant effects on soil fungal communities, but there was a significant interaction between the plant and time (Table 1). The ANOSIM also showed significant differences for soil bacterial community (*r* = 0.602, *p* = 0.002) between *A. kalkora* and *L. leucocephala* after 80 days, but no significant differences for the fungal community between *A. kalkora* and *L. leucocephala* after 80 days (*r* = 0.078, *p* = 0.165) as well as 50 days (*r* = 0.481, *p* = 0.098).

**Table 1:** Results of PERMANOVA analysis for rhizosphere soil bacterial and fungal community in laboratory cultivation experiment.

Soil microbe	Factor	df	*R* ^2^	*F*	*p*
Rhizosphere bacterial community	Plant	1	0.080	2.552	0.008
	Time	2	0.265	4.201	0.001
	Plant×time	2	0.087	1.384	0.064
Rhizosphere fungal community	Plant	1	0.049	1.380	0.154
	Time	2	0.138	1.944	0.020
	Plant×time	2	0.176	2.486	0.005

### 3.3 Rhizosphere soil bacterial community composition of the two legumes

**Figure 5:**
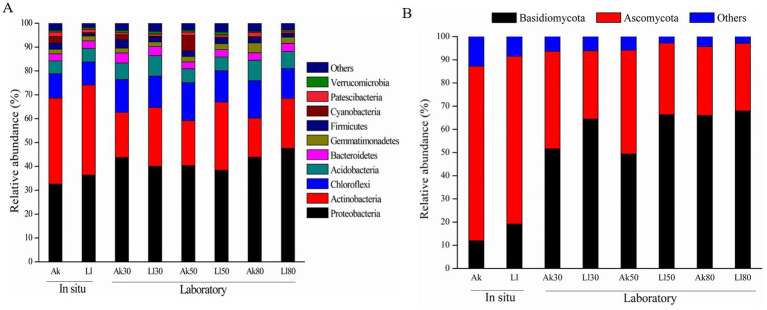
Effect of plant treatments on the relative abundance of soil bacterial **(A)** and fungal **(B)** communities at the phylum level. Ak, *Albizia kalkora*; Ll, Leucaena leucocephala; Ak30: cultivate by *Albizia kalkora* after 30 days; Ak50: cultivate by *A. kalkora* after 50 days; Ak80: cultivate by *A. kalkora* after 80 days; Ll30: cultivate by *Leucaena leucocephala* after 30 days; Ll 50: cultivate by *L. leucocephala* after 50 days; Ll 80: cultivate by *L. leucocephala* after 80 days.

**Figure 6:**
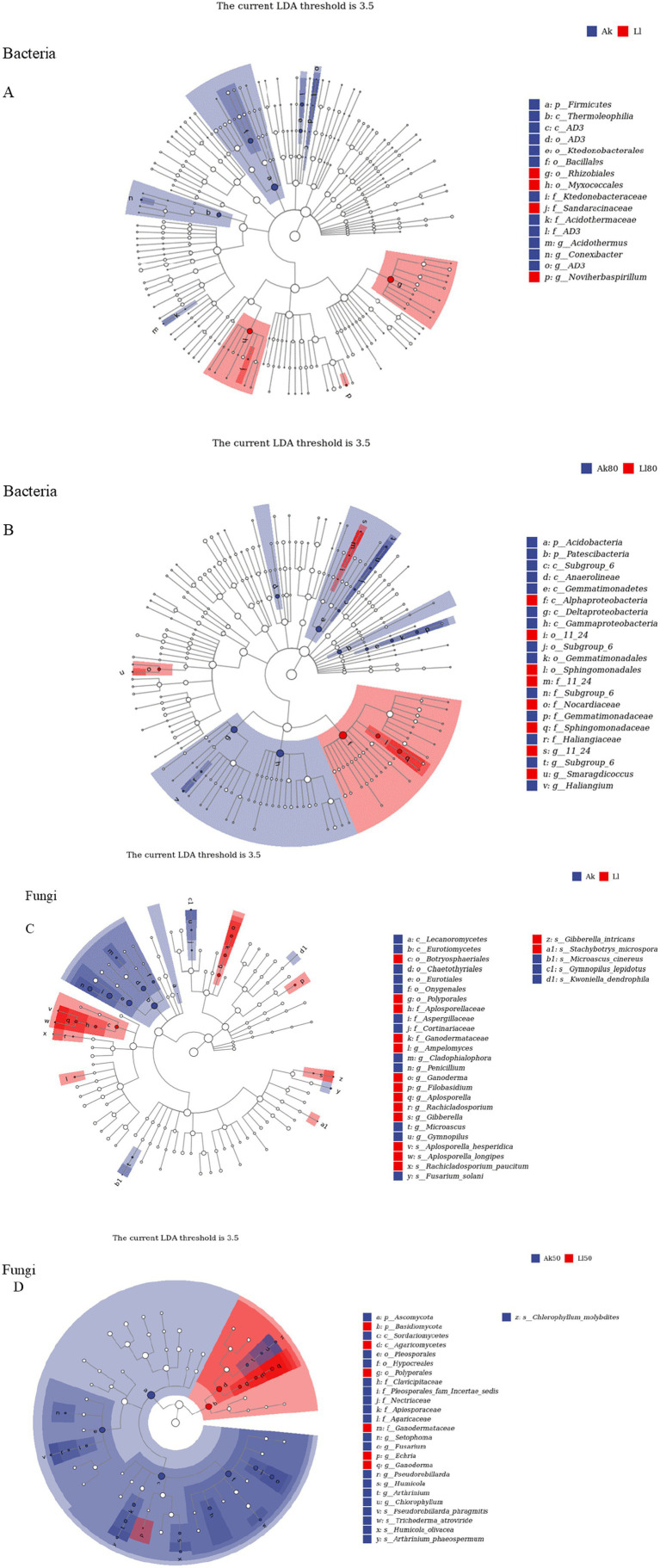
LDA score of the LEfSe analysis for rhizosphere soil bacterial **(A,B)** and fungal **(C,D)** community. Enriched taxa from the phylum to the species level with an LDA score >3.5 were shown in the histogram. Ak, *Albizia kalkora*; Ll, Leucaena leucocephala; Ak30: cultivate by *Albizia kalkora* after 30 days; Ak50: cultivate by *A. kalkora* after 50 days; Ak80: cultivate by *A. kalkora* after 80 days; Ll30: cultivate by *Leucaena leucocephala* after 30 days; Ll 50: cultivate by *L. leucocephala* after 50 days; Ll 80: cultivate by *L. leucocephala* after 80 days.

The dominant phyla in the bacterial community were Proteobacteria, Actinobacteria, Chloroflexi, Acidobacteria, Bacteroidetes, Gemmatimonadetes, Firmicutes, Cyanobacteria, Patescibacteria, and Verrucomicrobia, accounting for > 95% of the total bacterial sequences. The most dominant phyla were Proteobacteria and Actinobacteria, with the relative abundance remaining >50% (Figure 5A). LEfSe analysis showed (Figure 6A) that 16 biomarkers in rhizosphere soil bacterial community had significant differences between *A. kalkora* and *L. leucocephala in situ* sampling (*p* < 0.05, LDA > 3.5). Among the taxonomic groups with significant differences, four biomarkers were higher in the soil of *L. leucocephala*, and all of which belonged to Proteobacteria. Firmicutes was higher in *A. kalkora* soil than that in *L. leucocephala* soil. 22 biomarkers in rhizosphere soil bacterial community had significant differences between the two legumes after 80 days under cultivation conditions (Figure 6B), and o_Sphingomonadaceae, g_11_24, and g_ Smaragdicoccus were higher in the soil of *L. leucocephala* than those of *A. kalkora*. Besides, Acidobacteria and Patescibacteria were higher in *A. kalkora* soil than those in *L. leucocephala* soil.

### 3.4 Rhizosphere soil fungal community composition of the two legumes

For rhizosphere soil fungal community, Basidiomycota and Ascomycota were the dominant phyla, accounting for about 90% of the relative abundance (Figure 5B). The relative abundance of other fungal phyla was about 5%, including a small amount of Mortierellomycota, Chytridiomycota, and Glomeromycota. 30 biomarkers had significant differences in the fungal community between *A. kalkora* and *L. leucocephala in situ* sampling (*p* < 0.05, LDA > 3.5; Figure 6C). Among them, s_Fusarium solani was lower in *L. leucocephala* soil than that in *A. kalkora* soil. Under cultivation conditions, only two biomarkers (f_Hypocreaceae, s_Trichoderma_atroviride) had significant differences in the fungal community after 80 days, both with higher abundance in *A. kalkora* soil than those in *L. leucocephala* soil; however, 26 biomarkers differed significantly between the two plant species after 50 days (Figure 6D).

### 3.5 Functional prediction of rhizosphere microbial communities

**Figure 7:**
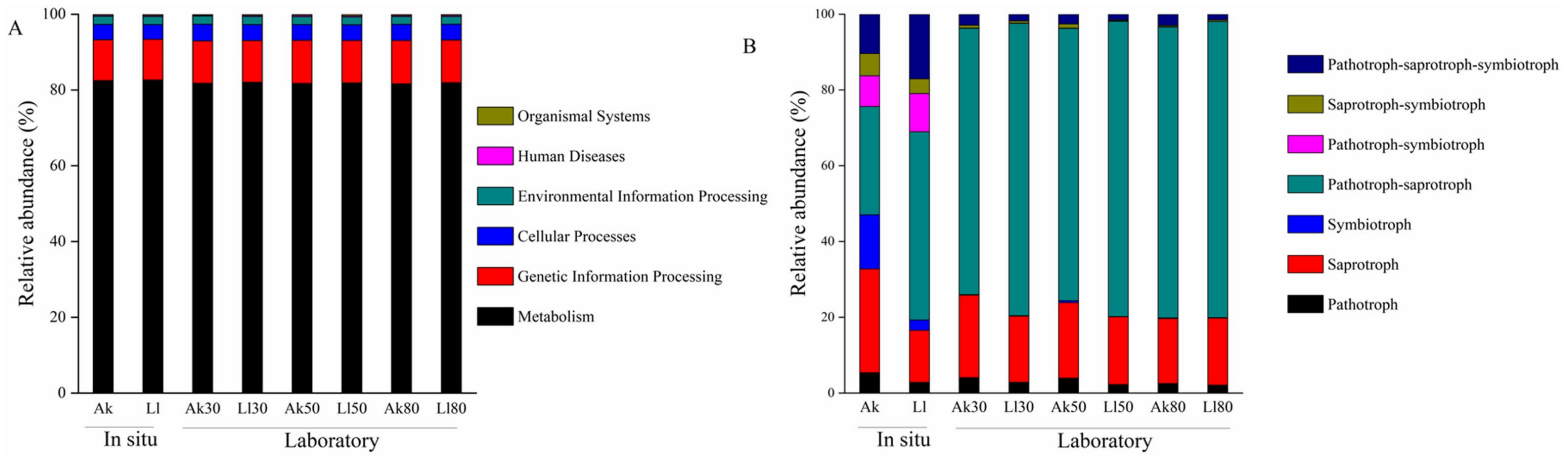
The rhizosphere bacterial community functions predicted by PICRUSt2 **(A)** and the fungal community functions predicted by FUNGuild **(B)** under different treatments. Ak, *Albizia kalkora*; Ll, Leucaena leucocephala; Ak30: cultivate by *Albizia kalkora* after 30 days; Ak50: cultivate by *A. kalkora* after 50 days; Ak80: cultivate by *A. kalkora* after 80 days; Ll30: cultivate by *Leucaena leucocephala* after 30 days; Ll 50: cultivate by *L. leucocephala* after 50 days; Ll 80: cultivate by *L. leucocephala* after 80 days.

Bacterial community functions were predicted using PICRUSt2 against the KEGG database. Across all treatments, six primary metabolic pathways (Level 1) were identified, among which metabolism consistently exhibited the highest relative abundance (approximately 80%), followed by genetic information processing, cellular processes, and environmental information processing (Figure 7A). No significant differences in the relative abundance of these six primary pathways were detected between *A. kalkora* and *L. leucocephala*, regardless of *in situ* or cultivation conditions. A total of 37 secondary metabolic pathways (level 2) were identified, 17 of which had a relative abundance exceeding 1%. Under in situ sampling, no significant differences were observed between the two plant species. After 80 days of cultivation, the relative abundance of amino acid metabolism of *L. leucocephala* was 1.33% higher than that of *A. kalkora*, with the corrected *p*-value indicated a marginally significant difference (*p* < 0.1).

FUNGuild detected all three main trophic modes: saprotroph, pathotroph, and symbiotroph. Several complex trophic modes were observed, and pathotroph-saprotroph was the primary type (Figure 7B). Saprotroph and Symbiotroph in the soil of *A. kalkora* were significantly higher than those of *L. leucocephala in situ* sampling. No significant differences were found for the three main trophic modes under cultivation conditions, but within the symbiotroph, the abundance of AMF in the soil of *L. leucocephala* was significantly higher than that of *A. kalkora* (Supplementary Figure S1).

### 3.6 The relationship between soil properties and rhizosphere microbial communities

**Figure 8:**
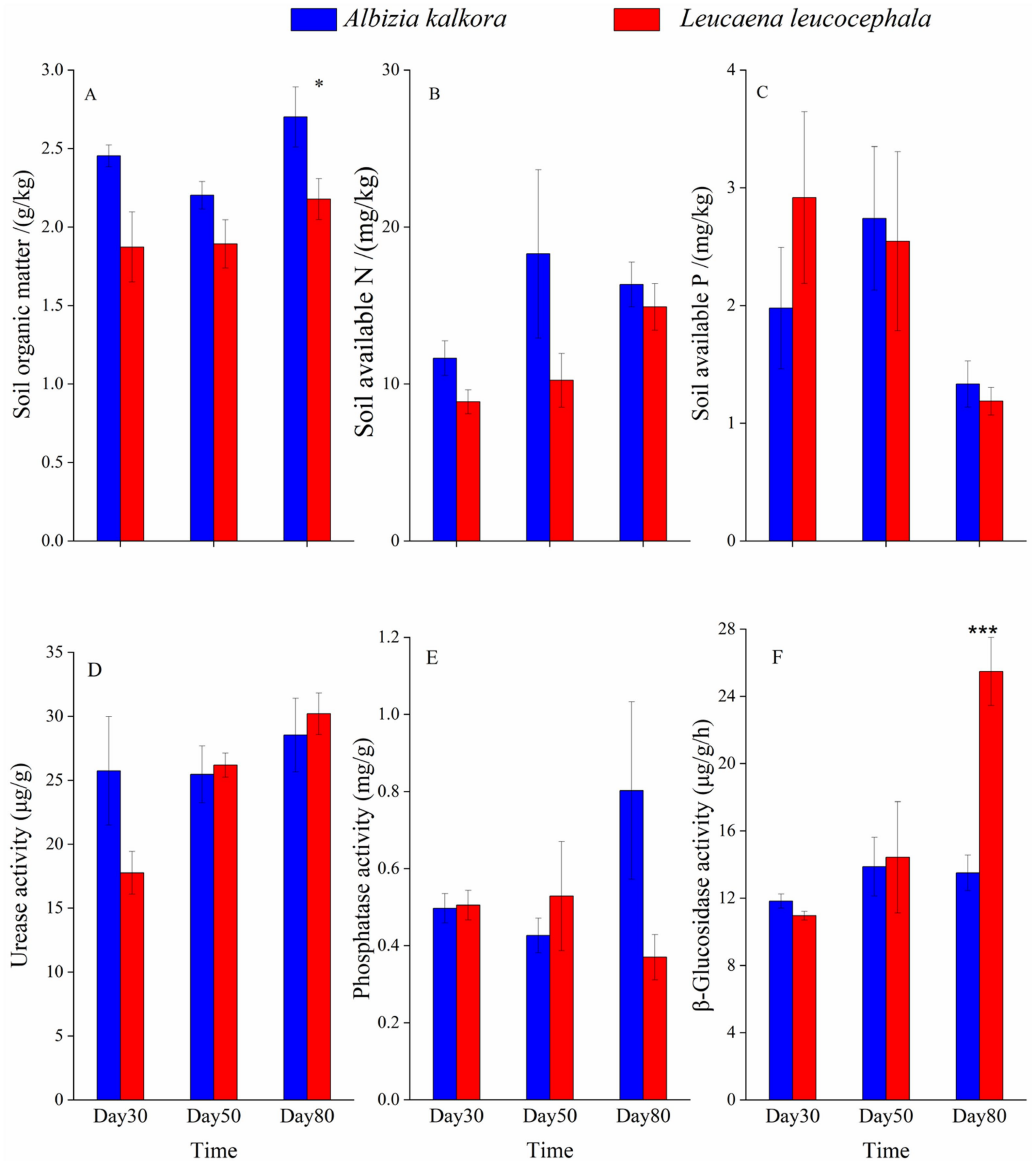
Effect of plant treatments on soil organic matter **(A)**, available N **(B)**, available P **(C)**, urease activity **(D)**, acid phosphatase activity **(E)**, and β-1, 4-glucosidase activity **(F)** under different cultivation time. *p*-values were derived from the independent-samples *t*-test. *p < 0.05, **p < 0.01, ***p < 0.001.

**Figure 9:**
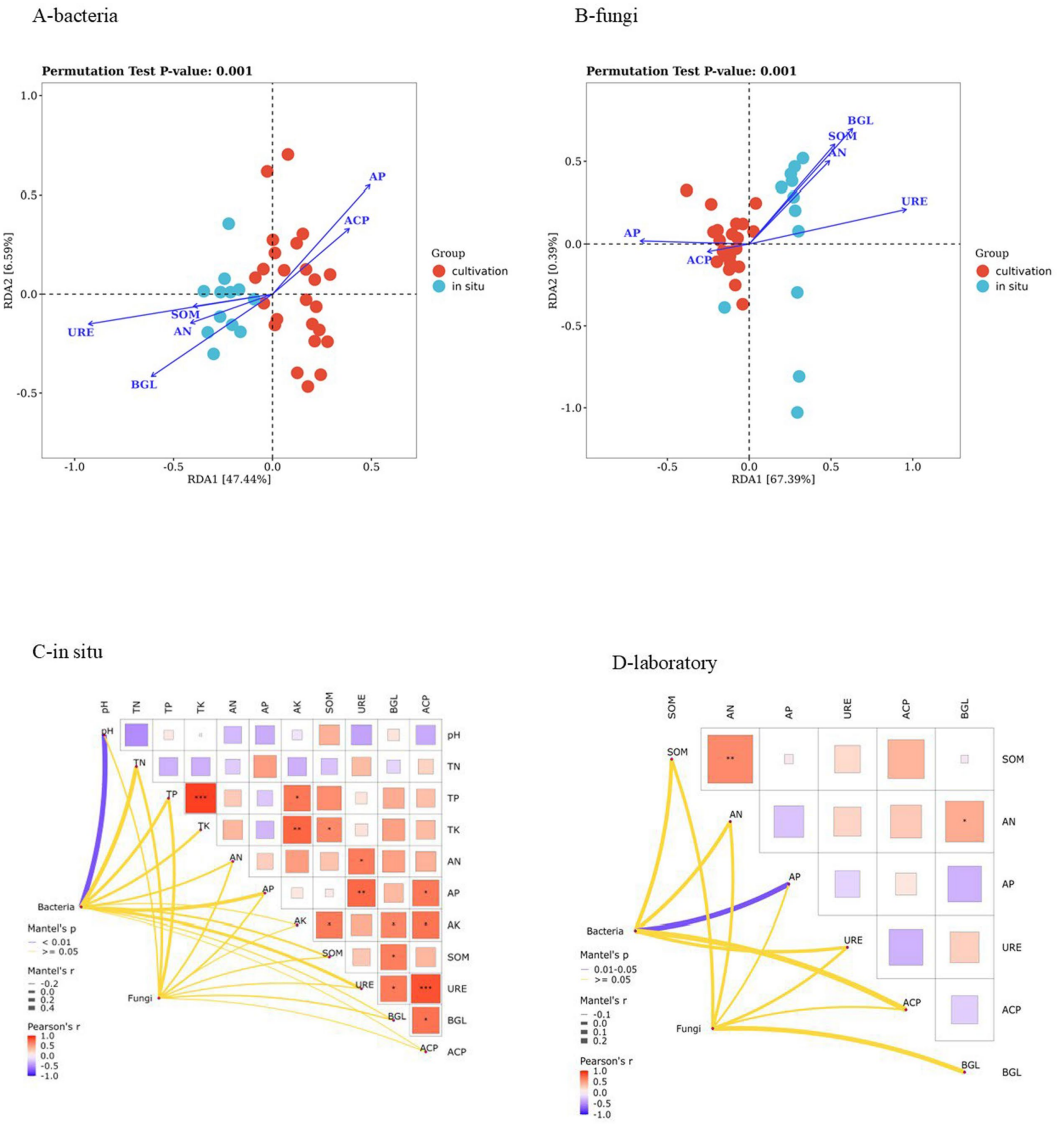
Relationships of rhizosphere soil microbial community and soil properties. **(A)** Redundancy analysis (RDA) of rhizosphere soil bacterial community and soil properties; **(B)** RDA of rhizosphere soil fungal community and soil properties; **(C)** Mantel tests of differences in soil bacteria with soil properties; **(D)** Mantel tests of differences in soil fungi with soil properties. pH, soil pH; TN, total nitrogen; TP, total phosphorus; TK, total potassium; AN, available nitrogen; AP, available phosphorus; AK, available potassium; SOM, soil organic matter; URE, urease activity; BGL, β-1, 4-glucosidase activity; ACP, acid phosphatase activity.

Detailed edaphic properties of the soil samples *in situ* sampling were provided in Table 2, and all these measured soil parameters showed no significant differences between *A. kalkora* and *L. leucocephala* soils (*p* > 0.05). Under cultivation conditions, available P content decreased with time, and *A. kalkora* soil had 24.57% higher organic matter and 46.97% lower β-glucosidase activity than *L. leucocephala* soil after 80 days (Figure 8). Redundancy analysis (RDA) revealed a significant correlation between soil properties and microbial composition (Figures 9A,B). The first two axes explained 54.03 and 67.78% of the total variance in bacterial and fungal community composition, respectively. Among the soil properties, urease (36.8%) and β-glucosidase activities (20.7%) accounted for the highest proportion of explained variance, followed by available P (18.5%) and phosphatase activity (9.4%). Similarly, urease and β-glucosidase activities exhibited the strongest correlations with fungal community composition, with available P, organic matter, and available N also showed significant correlations. Urease and β-glucosidase activities were mainly associated with *in situ* microbial communities, whereas available P was primarily associated with the cultured microbial communities. Under the same conditions, the Mantel test revealed that soil pH was the only determinant of differences for soil bacterial communities under in situ conditions (*p* < 0.01). In contrast, differences in soil fungal communities were not significantly affected by soil properties (Figure 9C). Under cultivation conditions, the bacterial community was significantly correlated with soil available P, and fungal community again showed no significant correlation with soil properties (Figure 9D).

**Table 2:** Soil parameter information of *A. kalkora* and *L. leucocephala* in the sampling sites and the statistical results of independent-samples T test.

Soil parameters	*A. kalkora*	*L. leucocephala*	*p*
pH	6.31 ± 0.25	6.65 ± 0.07	0.221
Organic matter/g·kg^−1^	5.48 ± 1.18	3.16 ± 0.50	0.102
Total N/g·kg-1	0.39 ± 0.03	0.40 ± 0.02	0.817
Total P/g·kg^−1^	0.12 ± 0.01	0.12 ± 0.01	0.448
Total K/g·kg^−1^	6.92 ± 0.35	6.92 ± 0.35	0.054
Available N/mg·kg^−1^	24.36 ± 4.07	17.59 ± 1.74	0.159
Available P/mg·kg^−1^	0.19 ± 0.05	0.16 ± 0.05	0.688
Available K/mg·kg^−1^	77.58 ± 12.54	59.38 ± 5.59	0.260
Urease/μg·g^−1^	188.58 ± 28.30	179.59 ± 17.02	0.790
β-Glucosidase/μg·g^−1^·h^−1^	45.27 ± 12.23	45.25 ± 10.26	0.999
Acid phosphatase/mg·g^−1^	0.46 ± 0.10	0.30 ± 0.04	0.161

Values in the table are means ± standard error (*n* = 6).

## 4 Discussion

### 4.1 Distinct rhizosphere microbial communities between native *A. kalkora* and exotic *L. leucocephala*

Our study revealed significant differences in rhizosphere soil microbial communities between *A. kalkora* and *L. leucocephala* under both *in situ* and cultivation conditions. *L. leucocephala* exhibited lower bacterial diversity than *A. kalkora*, and its fungal diversity was also lower after 50 days of cultivation (Figures 3, 4), supporting Hypothesis 1. Under in situ conditions, the relative abundances of Firmicutes and Fusarium were significantly lower in *L. leucocephala* soil than those in *A. kalkora* soil (Figure 6). Reduced Firmicutes abundance has also been reported in soil associated with the invasive *Flaveria bidentis* (Song et al., 2017). As Fusarium species are among the most important phytopathogenic and toxigenic fungi (Ma et al., 2010), their lower abundance suggests that *L. leucocephala* may escape from certain local pathogenic fungi, a finding that aligns with the enemy release hypothesis for alien plant species (Busby et al., 2016; McGinn et al., 2018). Under cultivation conditions, the relative abundances of Acidobacteria and Patescibacteria were significantly lower in *L. leucocephala* soil than those in *A. kalkora* soil. However, Firmicutes, which differed significantly under *in situ* conditions, showed no significant difference under cultivation conditions, indicating that field-observed differences do not necessarily re-emerge under cultivation conditions, contrary to hypothesis 2. A lower abundance of Acidobacteria has also been observed in soil associated with the invasive *Rhus typhina* (Zhu et al., 2020). Acidobacteria are typically more abundant in soils with higher organic carbon content (Yao et al., 2018). After 80 days of cultivation, soil organic matter of *A. kalkora* was higher than that of the *L. leucocephala* (Figure 8), which may promote the proliferation of Acidobacteria.

Invasive plant species tend to reduce soil microbial diversity compared with native species (Gaggini et al., 2018; Jurkšienė et al., 2020; Zhu et al., 2020), a pattern fully supported by our observations. Under both in situ and cultivation conditions, bacterial alpha diversity was significantly lower in *L. leucocephala* soil than that in *A. kalkora* soil. Invasive plants may reduce soil microbial diversity by selectively recruiting beneficial taxa while simultaneously suppressing others (Busby et al., 2016; Berlow et al., 2024). Additionally, *L. leucocephala* is allelopathic and produces certain allelochemicals that may be released into the rhizosphere soil (Kato-Noguchi and Kurniadie, 2022), directly inhibiting sensitive microbial groups and lowering diversity. Conversely, the more diverse microbial communities in *A. kalkora* soil are expected to have a greater likelihood of including less beneficial symbionts, which may proliferate and reduce the average efficiency of microbial mutualism (Bever et al., 2013). Unlike bacterial diversity, fungal alpha diversity did not differ significantly between species under in situ conditions or after 80 days of cultivation, but did differ after 50 days (Supplementary Table S3). Fungi are more susceptible than bacteria to plant interactions, with plant species-specific effects driving soil fungal communities (Hannula et al., 2019; Li et al., 2021), suggesting that rhizosphere fungal communities exhibit stronger host sensitivity. The lack of divergence in fungal alpha diversity after 80 days and under in situ conditions likely reflects strong soil available P limitation, as soil available P content was very low in both settings. P availability is a primary determinant of fungal community assembly (Wu et al., 2022). When soil P is not limiting, host plants exert stronger selective pressure, driving differentiation in fungal diversity. Under P-limited conditions, however, environmental filtering may override host effects, leading to convergent fungal communities dominated by stress-tolerant functional guilds.

### 4.2 Potential drivers of rhizosphere soil microbial community

The rhizosphere is recognized as a hub of microbial activity and one of the most intricate ecosystems, with its microbial community structure influenced by multiple factors (Chukwuneme and Babalola, 2025). According to the amplification-selection model (Wang et al., 2020), rhizosphere microbiota arises from the substantial amplification of microorganisms recruited from bulk soil. Recent studies have shown that local soils serve as the primary source of rhizosphere bacterial communities, homogenizing the community composition among different plant species growing at the same site, reflecting the unique interactions between plants and their surrounding bulk soil microorganisms (Li et al., 2019; Guajardo-Leiva et al., 2022). In our field investigation, *A. kalkora* and *L. leucocephala* grew sympatrically at the same site with no significant differences in soil physicochemical properties or enzyme activities. Thus, the bulk soil microbial communities beneath the two plant species were likely highly similar. However, because we did not characterize non-rhizosphere bulk soil, we cannot fully exclude the possible influence of the background microbiota on rhizosphere communities. To address this limitation, we conducted a laboratory cultivation experiment using homogenized soil to standardize the initial microbial background for both plant species. Our results confirmed that the two plants harbored distinctly different rhizosphere microbial communities.

Plants shape rhizosphere microbial communities through direct selective pressure (e.g., root exudation and symbiosis) and indirect modification of soil resource regimes (Bhatnagar et al., 2018; Hu et al., 2018; Bais et al., 2006). It has been shown that most of the groups (more than 96.5%) in the rhizosphere microbiome were determined by soil properties, and the genetic-dominated microbiome accounted for a small proportion (less than 3.5%; Xun et al., 2024). Therefore, plants and soil cooperatively shape the structure and function of microbial communities in the rhizosphere (Berg and Smalla, 2009; Liu et al., 2020). In this study, soil bacterial communities were found to be associated with soil properties under both *in situ* sampling and cultivation conditions (Figure 9), partially supporting hypothesis 3. Although soil pH did not differ significantly between the two legumes under *in situ* conditions, it was significantly correlated with bacterial community composition, consistent with previous reports showing strong links between pH and dominant bacterial taxa (Yao et al., 2018; Lopes et al., 2021). Typically, bacteria exhibit optimal growth within a narrow pH range of 6.7 to 7.5. Accordingly, in this study, the soil bacterial community was also correlated with pH even within such a limited range. Besides, the ecosystem of dry hot valley was limited by soil available P, and the abundance of Actinomycetes and fungi showed significant positive relationships with soil P fractions in this area (Lin et al., 2019; Jin et al., 2024). With increasing cultivation time, soil available P content decreased, thereby restricting bacterial growth and development. Consequently, under cultivation conditions, the soil bacterial community was correlated with soil available P. In contrast, fungal communities showed no significant correlation with soil properties under either in situ or cultivation conditions, consistent with findings from Huang et al. (2023). Host plant species exert a specific effect on fungal recruitment (Guajardo-Leiva et al., 2022). Given the stronger host specificity of fungi (Hannula et al., 2019; Li et al., 2021), the fungal community may be primarily related to root exudates rather than soil properties.

The two experimental conditions produced distinct soil properties. Compared to in situ conditions, the cultivation system exhibited significantly lower soil organic matter, available N, urease activity, and β-glucosidase activity, but significantly higher available P and phosphatase activity (Figure 8; Table 2). The physical sieving process likely reduces soil organic matter by removing light organic fractions and promoting organic C mineralization (Gao et al., 2017). The reduction of soil organic matter, in turn, decreases available N, urease, and β-glucosidase by reducing substrates for N mineralization and C-degrading enzymes (Loeppmann et al., 2016). In contrast, higher available P and phosphatase activity reflects P limitation, which drives phosphatase activity and consequently enhances available P release (Wang et al., 2018). These soil property shifts likely shaped distinct rhizosphere microbial assemblages (Figures 3–5), explaining why cultivation conditions did not fully reproduce the community compositions observed under field conditions. For both bacteria and fungi, urease and β-glucosidase activities showed the strongest correlations with microbial community composition, indicating that rhizosphere microbial dynamics are primarily coupled with C and N cycling processes (Huang et al., 2025; Yin et al., 2025). β-Glucosidase activity is highly sensitive to soil management and experimental treatments, and thus serves as a reliable indicator for assessing soil quality and health (Caldwell et al., 1999; Knight and Dick, 2004). Interestingly, β-glucosidase activity was generally lower in cultivated soils than *in situ* field soils, yet it was significantly higher in *L. leucocephala* soil than in *A. kalkora* soil under cultivation, highlighting that soil disturbance and microenvironmental alteration strongly modulate microbial C-cycling functioning and plant species effects. In the rhizosphere, root exudates represent the primary and most labile C source for heterotrophic microorganisms, with elevated labile organic C driving increased *β*-glucosidase activity (Zhou et al., 2024). Under cultivation conditions, the overall lower β-glucosidase activity likely reflects a reduction in labile root-derived carbon inputs due to the short plant growth period. In contrast, *L. leucocephala* soil exhibited significantly higher β-glucosidase activity than *A. kalkora* soil, suggesting a species-specific strategy. Consistent with a recent study showing that *L. leucocephala* successfully acquires nutrients by investing in below-ground biomass (Ndabankulu et al., 2022), *L. leucocephala* may allocate more recent photosynthetic C to the rhizosphere as root exudates, thereby “priming” or “mining” the decomposition of stable soil organic matter to acquire nutrients. The lower soil organic matter content in *L. leucocephala* soil compared to *A. kalkora* soil after 80 days of cultivation (Figure 8) is consistent with the “C-mining” strategy, resulting in a net loss of soil organic C over the short term.

### 4.3 Possible mechanisms for *L. leucocephala* invasion

Invasive plants can alter soil microbial communities and may generate positive plant–soil feedback that enhances their performance (Gaggini et al., 2018; Duchesneau et al., 2021). However, it remains unclear whether shifts in microbial community structure are a cause or a consequence of plant invasion (Bickford et al., 2020). Our study demonstrated that alteration of rhizosphere microbial communities by *L. leucocephala* invasion is a consequence; whether these changes facilitate its invasion requires further plant–soil feedback experiments. Under both *in situ* and cultivation conditions, we consistently observed significantly lower bacterial diversity in *L. leucocephala* rhizosphere soil compared with *A. kalkora*. Given the long co-evolutionary history between native *A. kalkora* and soil microorganisms, a broader range of soil microorganisms may co-exist with *A. kalkora*. This “high diversity” strategy may enhance the functional redundancy and stability of its symbiotic microbiome (Taylor et al., 2020), increasing the host’s capacity to cope with fluctuating or stressful environmental conditions, a potentially advantageous trait in the resource-limited and seasonally dry-hot valley ecosystem.

In contrast, the exotic *L. leucocephala* may adopt a distinct “low diversity-high efficiency” strategy by detecting and selecting some highly efficient soil microorganisms to promote its growth. Under cultivation conditions, when facing soil disturbance, *L. leucocephala* exhibited higher AMF abundance, *β*-glucosidase activity, and amino acid metabolism. By detecting, discriminating, and rewarding beneficial AMF partners (Toby Kiers et al., 2011; Werner et al., 2014), invasive plants can alter soil AMF communities to generate positive feedback that enhances their performance (Van der Putten et al., 2007), especially under low-P conditions (Zhang et al., 2010; Chen et al., 2020). Plant invasion also stimulates soil enzymes involved in C, N and P cycling, with the most pronounced enzymatic responses occurring during early invasion and when invaders form mycorrhizal associations (Chen et al., 2025; Negesse et al., 2025). In our study, the higher β-glucosidase activity and amino acid metabolism in *L. leucocephala* rhizosphere indicate greater microbial investment in N acquisition and organic matter decomposition. The early stage of plant establishment is critical for invasion success, and plant soil feedback for seedling establishment was an important predictor of invasive success (Aldorfová et al., 2020). Thus, although the 80-day cultivation captures only early rhizosphere assembly, the significant differences observed suggest that *L. leucocephala* can rapidly establish a distinct rhizosphere microbial community that may confer early competitive advantages.

## 5 Conclusion

In conclusion, this site-specific investigation of a native and an invasive legume revealed significant differences in rhizosphere soil microbial communities between *L. leucocephala* and *A. kalkora*. At the studied site, *L. leucocephala* exhibited lower rhizosphere bacterial diversity, along with reduced relative abundances of Firmicutes and Fusarium solani, compared with the native *A. kalkora*. After 80 days of cultivation, bacterial communities changed significantly, while fungal community composition and diversity remained similar between the two species. Under cultivation condition, *L. leucocephala* displayed lower abundances of Acidobacteria and Patescibacteria but higher abundances of AMF, amino acid metabolism, and β-glucosidase activity. The rhizosphere bacterial community correlated with soil pH under in situ conditions and with available P under cultivation, whereas fungal communities showed no significant correlation with soil properties, suggesting they were more shaped by plant-specific root exudates. Collectively, this study characterizes distinct rhizosphere microbial assemblages associated with the two legumes at a co-established site and during early stage in the cultivation experiment. Future work is required to explore the potential microbial feedback effects that modulate plant growth and competition.

## Data Availability

The original contributions presented in the study are publicly available. This data can be found at: National Center for Biotechnology Information (NCBI) Sequence Read Archive (SRA) database under BioProject PRJNA1481909.

## Footnotes

1
http://huttenhower.sph.harvard.edu/lefse/

2
https://github.com/UMNFuN/FUNGuild
