# Supplementary material for: Exotic Leucaena leucocephala cultivates distinct rhizosphere soil microbial communities from native Albizia kalkora in a dry-hot valley of China
Source: Front Microbiol. 2026 Jul 8;17:1866484. doi: 10.3389/fmicb.2026.1866484 (PMC13388394; doi:10.3389/fmicb.2026.1866484)
Supplement: Supplementary file 1 [file Data_Sheet_1.docx]

Table S1 The growth information of *A. kalkora* and *L. leucocephala* under in situ sampling

| Plant | Height (m) | Diameter (cm) |
| --- | --- | --- |
| *A. kalkora*-1 | 2 | 4 |
| *A. kalkora*-2 | 6 | 12 |
| *A. kalkora*-3 | 0.5 | 3 |
| *A. kalkora*-4 | 1.2 | 3.2 |
| *A. kalkora*-5 | 0.8 | 1.7 |
| *A. kalkora*-6 | 0.2 | 2 |
| *L. leucocephala*-1 | 2.5 | 2 |
| *L. leucocephala*-2 | 1.95 | 1 |
| *L. leucocephala*-3 | 3.6 | 4 |
| *L. leucocephala*-4 | 2.76 | 3 |
| *L. leucocephala*-5 | 1.65 | 2 |
| *L. leucocephala*-6 | 2.1 | 2.5 |

Table S2 Plant growth characteristics at different time and statistical results of two-way ANOVA under cultivation condition

| Time | Plant | Height (cm) | Root weight (g) | Above mass (g) | Litter mass (g) | Nodule numbers | Nodule weight (g) |
| --- | --- | --- | --- | --- | --- | --- | --- |
| 30 days | *A. kalkora* | 9.16±0.76 | 0.23±0.01 | 0.4±0.03 | 0±0 | 27±8.08 | —— |
|  | *L. leucocephala* | 10.43±1.04 | 0.26±0.03 | 0.37±0.02 | 0.03±0.01 | 7±1.15 | —— |
| 50 days | *A. kalkora* | 10.73±0.79 | 1±0.1 | 0.82±0.07 | 0.02±0.01 | 59±13.45 | —— |
|  | *L. leucocephala* | 14.4±0.81 | 0.84±0.14 | 0.71±0 | 0.06±0 | 28.67±5.17 | —— |
| 80 days | *A. kalkora* | 13.55±0.43 | 1.86±0.29 | 1.29±0.1 | 0.1±0.02 | 32.17±5.06 | 0.16±0.03 |
|  | *L. leucocephala* | 18.66±0.37 | 2.22±0.1 | 1.37±0.02 | 0.25±0.04 | 81.83±7.62 | 0.25±0 |
| Plant | | <0.001 | 0.696 | 0.704 | 0.016 | 0.973 | 0.056 |
| Time | | <0.001 | <0.001 | <0.001 | <0.001 | <0.001 | —— |
| Plant×time | | 0.021 | 0.445 | 0.414 | 0.142 | <0.001 |  |

Table S3 The *P* values for alpha diversity indices of soil bacterial and fungal community based on Mann-Whitney U test with Benjamini‑Hochberg correction

| Soil microbe | Alpha diversity | In situ | Cultivation conditions | | |
| --- | --- | --- | --- | --- | --- |
|  |  |  | 30 days | 50 days | 80 days |
| Soil bacterial community | Chao1 | 0.200 | 0.827 | 0.827 | 0.873 |
|  | Evenness | 0.050 | 0.827 | 0.827 | 0.073 |
|  | Shannon | 0.050 | 0.827 | 0.827 | 0.073 |
|  | Simpson | 0.423 | 0.827 | 0.508 | 0.073 |
| Soil fungal community | Chao1 | 0.998 | 0.684 | 0.050 | 1 |
|  | Evenness | 0.998 | 0.684 | 0.050 | 1 |
|  | Shannon | 1 | 0.684 | 0.050 | 1 |
|  | Simpson | 0.998 | 0.827 | 0.050 | 1 |


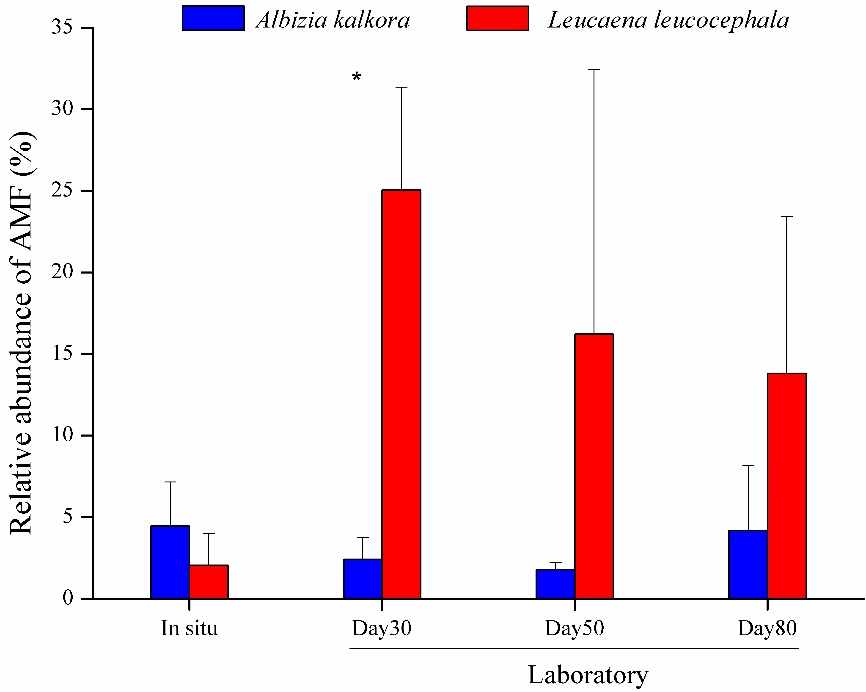


Fig. S1 The relative abundance of arbuscular mycorrhizal fungi (AMF) within the symbiotroph under different treatments
